# Tuning the Cytokine Responses: An Update on Interleukin (IL)-4 and IL-13 Receptor Complexes

**DOI:** 10.3389/fimmu.2018.00888

**Published:** 2018-06-07

**Authors:** Ilkka S. Junttila

**Affiliations:** ^1^Cytokine Biology Research Group, Faculty of Medicine and Life Sciences, University of Tampere, Tampere, Finland; ^2^Department of Clinical Microbiology, Fimlab Laboratories, Tampere, Finland

**Keywords:** interleukin-4, signal transduction, STAT6, interleukin-4 receptor, cytokine signaling, allergic inflammation

## Abstract

Interleukin (IL)-4 and IL-13 are related cytokines that regulate many aspects of allergic inflammation. They play important roles in regulating the responses of lymphocytes, myeloid cells, and non-hematopoietic cells. In T-cells, IL-4 induces the differentiation of naïve CD4 T cells into Th2 cells, in B cells, IL-4 drives the immunoglobulin (Ig) class switch to IgG1 and IgE, and in macrophages, IL-4 and IL-13 induce alternative macrophage activation. This review gives a short insight into the functional formation of these cytokine receptors. I will discuss both the binding kinetics of ligand/receptor interactions and the expression of the receptor chains for these cytokines in various cell types; both of which are crucial factors in explaining the efficiency by which these cytokines induce intracellular signaling and gene expression. Work initiated in part by William (Bill) E. Paul on IL-4 some 30 years ago has now grown into a major building block of our current understanding of basic immunology and the immune response. This knowledge on IL-4 has growing clinical importance, as therapeutic approaches targeting the cytokine and its signal transduction are becoming a part of the clinical practice in treating allergic diseases. Just by reading the reference list of this short review, one can appreciate the enormous input Bill has had on shaping our understanding of the pathophysiology of allergic inflammation and in particular the role of IL-4 in this process.

## Introduction

Allergic inflammation is an inappropriately controlled inflammatory response with characteristic hallmarks of eosinophilia, elevated immunoglobulin (Ig)E-levels, increased mucus production, and typical cytokine/chemokine expression. Clinically, these basic pathophysiological mechanisms result in symptoms varying from mild skin rash (atopic dermatitis) and runny nose (allergic rhinitis) to life-threatening problems in breathing (allergic asthma). This inflammatory process from the very initiation is critically regulated by cytokines and chemokines. The cytokines regulate cellular responses on transcriptional level, while chemokines play a role in recruiting inflammatory cells to the sites on inflammation. One of the central cytokines regulating allergic inflammation is interleukin (IL)-4 and since its cloning, efforts targeting IL-4 have been made to decrease IL-4-induced inflammation. In part, these efforts have been slowed down by the receptor of IL-4, which is ubiquitously expressed and easily saturated by the ligand. In this minireview, I briefly discuss the receptor system of IL-4 that is also shared by IL-13, how it elicits signaling, and how it has been recently therapeutically targeted. I also highlight the enormous input of Bill Paul in this field; learning the story of IL-4 is not only about IL-4 but has also helped in unfolding more profound biological phenomenon in how T cells can dynamically respond to changes in environment to output an appropriate response.

## IL-4 and IL-13 Production

Interleukin-4 and IL-13 are the signature cytokines of the type II inflammatory response. They are key players in the inflammatory response triggered either by an invading parasite or allergen. The cellular sources of IL-4 and IL-13 have been studied extensively and along with CD4 T cells, basophils, eosinophils, mast cells, and NK T cells, appropriately stimulated ILC2 cells have the ability to produce IL-4 and IL-13 (1–9).

The genomic locus, where IL-4 and IL-13 are produced (along with IL-5), is called the Th2 cytokine locus, which is located on chromosome 5 in humans and on chromosome 11 in mice and is under the control of the locus control region (LCR) of the Rad 50 gene (10, 11). The LCR in CD4 T-cells is indispensable for the production of IL-4 and IL-13 *in vivo* (12). The production of the two cytokines is not identical though: IL-4 production is calcineurin dependent, whereas IL-13 production is only partially dependent on calcineurin (13). Upon the appropriate stimulation of the cells, the LCR of the Th2 cytokine locus is epigenetically modified to allow the access of transcription factors to the DNA and the subsequent transcription of these cytokines. This complex regulation was recently reviewed in detail (10). Interestingly and in line with findings in mice, a polymorphism in the murine equivalent of the DNase I hypersensitive site (RHS)7 in humans affects DNA methylation and gene expression at 5q31 and subsequently IgE levels on a population level (14).

## IL-4 Receptor System

When IL-4 or IL-13 is released from T cells, cells carrying the receptors for these cytokines will respond. For IL-4 and IL-13, the unique utilization of the STAT6 transcription factor in the signaling they elicit allows them to execute specific functions on different cell types; IL-4 is the regulator of lymphocyte functions (Th2 differentiation and B-cell IgG1 and IgE class switch), whereas IL-13 is an effector cytokine, regulating smooth cell muscle contraction and mucus production in the airway epithelium, for example, in allergic asthma (15). In addition to IL-4 and IL-13, one report has shown that at least in human cells, thymic stromal lymphopoietin (TSLP) can induce the tyrosine phosphorylation of STAT6 (16), TSLP signaling will be discussed in detail below.

**Figure 1:**
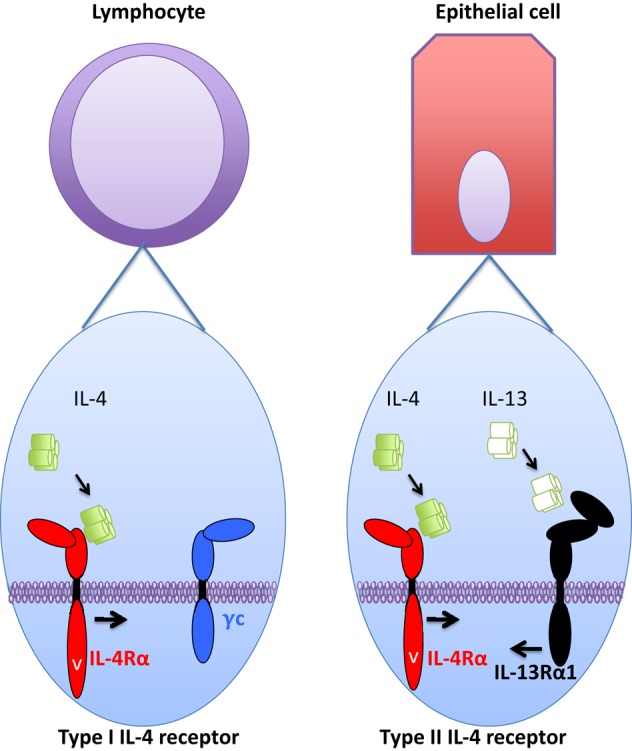
Type I and type II interleukin (IL)-4 receptor components and cellular distribution. Type I IL-4 receptor is mainly expressed in hematopoietic cells, and specifically in lymphocytes (left part) very little or no expression of type II receptor is observed. In non-hematopoietic cells, such as epithelial cells (right part), very little or no expression of type I IL-4 receptor is observed. Instead, type II IL-4 receptor is readily expressed and subsequently these cells are also responsive to IL-13 that utilizes type II IL-4 receptor, but “drives” it into opposite direction than IL-4. Myeloid cells (not pictured) fall in between these two cell types as they express both type I and type II IL-4 receptors.

The cytokine-binding receptor chain for IL-4 is IL-4Rα. This receptor chain is widely expressed, most cells carry at least low numbers of this receptor chain. Upon IL-4 binding to IL-4Rα, the IL-4/IL-4Rα-complex will bind a secondary receptor chain, either IL-2Rγc (γc) or IL-13Rα1 (Figure 1). The expression of these secondary chains varies among different cell types. In non-hematopoietic cells, γc expression is low or absent, whereas higher amounts of IL-13Rα1 are expressed in these cells. By contrast, lymphocytes express only low levels of IL-13Rα1 and relatively large amounts of γc. Finally, myeloid cells fall in between non-hematopoietic cells and lymphocytes, as they express of both IL-13Rα1 and γc.

Interleukin-4 and IL-13 regulate cellular functions and activate transcriptional machinery *via* cell surface receptors. For IL-4, binding of the cytokine to a single cell surface receptor chain (IL-4Rα) generates a ligand/receptor complex that requires the recruitment of a third receptor chain to form a functional receptor complex. The receptor formed by IL-4/IL-4Rα with γc is a type I IL-4 receptor and the IL-4/IL-4Rα complex binding IL-13Rα1 is a type II IL-4 receptor (17). Thus, based on their tissue distribution, the type I IL-4 receptor is found in lymphocytes and myeloid cells, and the type II IL-4 receptor is expressed in myeloid cells and all non-hematopoietic cells. The binding of IL-4 to IL-4Rα occurs with high affinity (Kd in the order of 10⋅10 M^−1^). This effectively means that at very low concentrations of IL-4 it can maximally occupy the receptor chains at a given cell surface.

It was originally assumed that the secondary recruitment of either γc or IL-13Rα1 into the IL-4/IL-4Rα dimer would occur with substantially lower affinity than the primary binding of IL-4 to IL-4Rα (18, 19). The expression levels of the secondary receptor chain would then become important. As the primary receptor chain for IL-4 is saturated easily, the formation of a functional receptor complex could be dictated by the availability of the second receptor chain (20). However, the initial binding measurements for the IL-4/IL-4Rα complex binding to γc or IL-13Rα1 were carried out in free solution. Cell membrane-bound γc and IL-13Rα1 behave differently in recruiting the IL-4/IL-4Rα complex under conditions of maximal ligand occupancy (21). While the recruitment of membrane-bound γc is relatively inefficient, the recruitment of IL-13Rα1 takes place roughly with the same efficiency as does the IL-13 driven IL-13/IL-13Rα1 binding to IL-4Rα (21). The authors suggested that early endosomes concentrated the receptor chains underneath the plasma membrane. However, if this is the case, it still remains unclear how IL-4 and IL-13 induce the phosphorylation of STAT6 differently in type I IL-4R-deficient macrophages from different locations, namely, BMDM and peritoneal cavity macrophages (20). If it is not the differential expression of IL-13Rα1 that explains the difference in the cytokine response between these macrophage populations, then a more profound difference in the IL-4Rα-induced STAT6 signaling pathway must be involved which remains uncharacterized. One plausible explanation might be differences in receptor endocytosis between the cells. For IL-13-induced type II IL-4 receptor signaling, IL-13 variants showing decreased IL-4Rα recruitment to the complex indicate that STAT6 signaling is regulated by receptor endocytosis (22). Quite recently, the role of the receptor transmembrane domain in regulating the recruitment of the type II IL-4 receptor has also become appreciated, and the cell type specific actin-dependent membrane microcompartments may participate in dictating the signaling potency of the type II IL-4R (23).

Once completely assembled, the IL-4 receptor complexes will induce intracellular signaling. The binding of IL-4 to the ectodomain of the IL-4Rα and subsequently to γc or IL-13Rα1, induces a conformational change in the intracellular receptor domains allowing the activation of intracellular signaling molecules. The Jak kinases, associated with γc (Jak3), IL-4Rα (Jak1), or IL-13Rα1 (Tyk2, Jak2), will auto- and cross-phosphorylate each other, resulting in their activation and the subsequent tyrosine (Y) phosphorylation of critical Y residues in IL-4Rα chain. Upon phosphorylation, the Y residues in the intracellular domains of IL-4Rα serve as docking sites for SH domains of intracellular signaling molecules (17). STAT6 and IRS molecules, in particular, become activated on these tyrosine residues in response to the activation of the type I IL-4 receptor. By contrast, the type II IL-4 receptor is unable to activate IRS significantly, whereas the activation of STAT6 occurs quite efficiently, which also means that IL-4 (*via* type I IL-4 receptor) activates IRS2 efficiently while IL-13 does not (24). Once activated, STAT6 molecules homodimerize and translocate to the nucleus where they bind specific accessible DNA sequences, for example, on the CD23 promoter in human B-cells and on the arginase1 enhancer in mouse macrophages (25, 26). IRS molecules do not translocate to the nucleus, but rather, they activate signaling pathways independent of STAT6 including PI3K, Akt, PKBE, and mTOR [reviewed in Ref. (27)].

In addition to signaling events that elicit transcriptional changes, pathways that negatively regulate activated signaling pathways are also upregulated by IL-4. Phosphatases, SOCS, and PIAS proteins all participate in the downregulation of the elicited signal, for detailed reviews on these inhibitory mechanisms, see Ref. (28, 29).

## IL-13 Receptor System

Like IL-4, IL-13 also has two receptors, but unlike IL-4, IL-13 utilizes two separate binding chains, namely, IL-13Rα1 and IL-13Rα2. Thus, the decision of whether a type I or a type II IL-4 receptor is formed occurs *after* the IL-4/IL-4Rα complex is formed, whereas IL-13 binding *upon* either IL-13Rα1 or IL-13Rα2 determines which receptor IL-13 utilizes. IL-13Rα2 binds IL-13 with higher affinity than IL-13Rα1. The role of IL-13Rα2 in IL-13 biology has been somewhat elusive, and it has been considered merely a decoy receptor that binds free IL-13 strongly, without eliciting signaling, and thus would serve as a “neutralizer” of IL-13, by efficiently internalizing IL-13 from extracellular spaces. Further studies on IL-13Rα2 have shown that the receptor chain is not only a decoy receptor. Indeed, Fichtner-Feigl and colleagues showed a role for IL-13Rα2-mediated signaling that required the cytoplasmic tail of IL-13Rα2 in the production of TGF-β1 providing evidence for IL-13Rα2-mediated signaling (30).

The IL-13Rα1-bound IL-13 “drives” the type II IL-4 receptor into the opposite direction, as does IL-4 (Figure 1). Thus, IL-13 binds IL-13Rα1, and the IL-13/IL-13Rα1 complex then recruits IL-4Rα into the functional receptor complex. The fully assembled receptor complex then activates the STAT6 transcription factor, but like IL-4 *via* the type II IL-4 receptor, IL-13 is a poor inducer of IRS activation through this receptor (24). The binding of IL-13 to IL-13Rα1 is relatively inefficient, indicating that once IL-13/IL-13Rα1 binding occurs, the ensuing formation of the functional receptor complex is likely. However, lowering the IL-13/IL-13Rα1-binding capability to IL-4Rα requires a substantial decrease in the second binding step to result in lowered STAT6 activation (22).

## IL-4- and IL-13-Induced Signaling: A Comparison of Signaling Induced by the Two Cytokines

Depending on the cell type, IL-4 and IL-13 both can activate STAT6 (Figure 1). As IRS2 is only weakly induced by type II IL-4 receptor [and thus IL-13; (24)], intracellular signaling elicited by the two cytokines is somewhat different. By inducing IRS2, IL-4 subsequently activates various pathways including Sos/Ras, PI3K/Akt, PKB/mTOR, or PKC [reviewed in Ref. (31)]. Of these pathways, mTOR has recently been linked to CD4 Th2 cell differentiation as well as alternative macrophage activation these results were recently thoroughly reviewed (32). Unfortunately, experimental therapeutic efforts targeting mTOR in murine allergic disease models have failed (33). Here, it is of note though that mTOR-based approaches target type I IL-4 receptor (i.e., IRS2 signaling), while many disadvantageous IL-4 effects, such as compromised epithelial barrier function, arise from IL-4 signaling *via* type II IL-4 receptor (34).

As pointed out earlier, lymphocytes respond poorly to IL-13. The expression of IL-4Rα (i.e., type I IL-4 receptor) plays thus a main role in lymphocyte responses to IL-4. The expression of IL-4Rα in naïve lymphocyte is relatively low and *in vitro*, a STAT5-dependent, STAT6-independent signal likely enhances IL-4Rα expression, which then in an autocrine manner, further upregulates IL-4Rα expression (35). Th2 cells then express large amounts of IL-4Rα and are further stimulated *via* IL-4. In case of Th1 or Th17 cells, the lack of IL-4-positive signal inhibits the upregulation of IL-4Rα, but in the case of Th1 cells, for example, the differentiation does not ablate the ability of the cells to respond to IL-4 (36). Interestingly, Th17 cells do express IL-13Rα1 (37).

For ILCs, the expression of IL-4 and IL-13 receptor(s) is still unclear. Several reports have established the ILC2-derived IL-13 acting on target cells *via* type II IL-4 receptor as a mechanism for several physiological functions such as beige fat biogenesis (38) or hepatic fibrosis (39) but if ILC2-derived IL-13 can act on autocrine manner has not been established. Future experiments will also be warranted to reveal if IL-4Rα is differently expressed between ILC subtypes to tune the cells either to IL-4 or IL-13.

## Therapeutic Utilization of the IL-4 Receptor System

The road for IL-4- and IL-4R-based treatments from bench to bedside has been a long and winding one (40). IL-4 has been considered a therapeutic target for boosting and redirecting T and B cell functions, but the usage of IL-4 itself has been problematic, not least due to the harmful side effects of activating the type II IL-4 receptor in non-hematopoietic cells (34). Furthermore, in mice, IL-4, but not IL-13, induced weight loss and spontaneous erythrophagocytosis (41). Theoretically, in this sense, an IL-4 that could activate only the type I IL-4 receptor but not the type II receptor could be advantageous. Structural studies of human IL-4 receptor complexes (18) indicated that once IL-4 is bound to IL-4Rα, the D-helix of IL-4 faces the secondary receptor chain and forms the interacting surface of IL-4/IL-4Rα to the second chain in question. This opened up opportunities to mutate the structure of the human IL-4 at the D-helix in a way that left the IL-4/IL-4Rα interaction intact but allowed the binding efficiencies of the IL-4/IL-4Rα complexes toward either γc or IL-13Rα1 to be altered. These studies indicated that a 1,000-fold induction in the recruitment of the IL-4/IL-4Rα complex to the secondary chain had surprisingly little effect on the immediate signaling induced by such an IL-4-mutant, as measured by STAT6 activation (42) and similar results were obtained with IL-13Rα1 bound IL-13 mutants with varying abilities to recruit IL-4Rα into the type II IL-4 receptor complex (22). However, in the case of the type I IL-4 receptor, when the availability of the second chain (γc) was decreased with a blocking antibody, the difference between the WT and the type I receptor-specific IL-4 mutant became more evident, suggesting that such IL-4 mutants could be used to redirect IL-4 responses into cells expressing small amounts of second chains for IL-4/IL-4Rα complexes (42).

When considering the harmful effects arising from excess IL-4 and IL-13, in for example allergies, knowledge of the structural and functional characteristics of the IL-4 receptors and their unique signaling *via* STAT6 has been useful in efforts to therapeutically modify IL-4/IL-13 biology. As an example of some therapeutic approaches used are indicated in Table 1. A set of monoclonal antibodies for blocking different aspect of the early events of IL-4 and IL-13 signaling are being considered for wider clinical use: dupilumab (43)—a monoclonal blocking antibody for IL-4Rα—lebrikizumab (44), anrukinzumab (45), tralokinumab (46)—blocking antibodies for IL-13—and pascolizumab—a blocking antibody for IL-4 (47) among others. Furthermore, pitrakinra, an IL-4 receptor antagonist that upon binding IL-4Rα, blocks both type I and type II IL-4 receptors has showed initial efficacy in clinical trials (48). The utilization of biological approaches to target IL-4/IL-13 pathways requires an understanding of the pathophysiological process underlying the inflammatory response. The cell type- and tissue-specific distribution of the IL-4/IL-13 receptor components adds to the complexity of the picture and probably in part explains this long and winding road of IL-4R system-based treatments from the initial cloning of the receptor and cytokines to the development of useful clinical applications. Interestingly, STAT6 inhibitor (AS1517499) has shown some potential in inhibiting prostate cancer cell growth [Table 1; (49)], which opens new possibilities in targeting the IL-4/IL-13 signaling therapeutically even beyond allergic diseases.

**Table 1:** Examples of various steps interleukin (IL)-4/IL-13 signaling could potentially be targeted.

Molecule name	Target molecule	Potential	Reference
Dupilumab	IL-4Ra	IL-4- and IL-13-mediated signaling	(43)
Pitrakinra	IL-4Ra	IL-4- and IL-13- mediated signaling	(48)
Leprikizumab	IL-13	IL-13-mediated signaling	(44)
Anrukinzumab	IL-13	IL-13-mediated signaling	(45)
Tralokinumab	IL-13	IL-13-mediated signaling	(46)
Pascolizumab	IL-4	IL-4-mediated signaling (trials aborted)	(47)
AS1517499	STAT6	IL-4- and IL-13-mediated signaling/transcription/proliferation in prostate cancer cells	(49)

## Another Shared Cytokine Receptor System: IL-7/TSLP

An analogous way of sharing cytokine receptor chains, as seen in the IL-4/IL-13 system, can be found in IL-7/TSLP receptor signaling. In this system, IL-7-bound-IL-7Rα binds γc and thus forms the complete IL-7 receptor, while TSLP binds TSLPR and then recruits IL-7Rα to the complex [reviewed in Ref. (50)]. Thus, theoretically, the IL-7/IL-7Rα/γc complex resembles the type I IL-4 and TSLP/TSLPR/IL-7Rα resembles the type II IL-4 receptor “driven” by TSLP. Furthermore, it is intriguing that TSLPR and γc are closely related structurally, sharing 24% identity to the common γ receptor chain (γc) (51, 52) with certain specific features associated with TSLPR as opposed to other type I cytokine receptors, including the PSxW(S/T) sequence cassette as opposed to WSxWS in the membrane proximal domain (53). However, this is where the analogy ends, as IL-7/IL-7Rα does not recruit TSLPR, but only γc to the receptor complex. Functionally, it seems that the IL-4/IL-13 receptor is “tuned” for differential purposes than is the IL-7/TSLP system. IL-4Rα is expressed ubiquitously and the second receptor chain (either γc or IL-13Rα1) is also widely distributed. Thus, IL-4 has access to virtually all cell types, and it can saturate receptors at low concentrations due to the efficient primary binding of IL-4 to IL-4Rα. For IL-13, the cytokine concentration required to saturate IL-13Rα1 needs to be higher as the binding efficiency of IL-13 binding to IL-13Rα1 is lower. In line with this, when PBMCs from atopic patients were stimulated with a mite allergen, the cells produced over 20 times more IL-13 than IL-4 (54). The notion of the “effector” function of IL-13 in, for example, parasite expulsion, combined with the known toxicity of IL-4, suggest that the system has evolved in a way that protects peripheral tissues from the toxicity of IL-4 by tuning the receptors in the periphery to be more responsive to IL-13 than to IL-4.

In the IL-7/TSLP system, the differential anatomical expression of the cytokines suggests that the sharing of the cytokine receptors might occur, because the cytokines are not expressed in same sites and thus would not limit the signaling of each other. Regulating the expression of just one receptor chain on the cell surface (IL-7Rα), will affect both. However, there are likely further lessons to be learned from TSLP and its functional receptors. Recently, neutrophils in mice were found to respond to TSLP (55), whereas at least in humans, neutrophils do not likely express IL-7Rα (56). It was recently also shown that dynamic IL-7Rα expression on DCs was required for IL-7 and TSLP responses (57), so one possibility might be that IL-7Rα is under very stringent regulation and is only upregulated in various cell types under very specific conditions.

## Concluding Remarks

Taken together, the organization and binding events of type I and type II IL-4 receptors have been reviewed here. The efficiency by which a functional IL-4/IL-13 receptor is formed appears to be a sum of three parameters. First, the binding efficiency of a cytokine to the cytokine-binding receptor chain dictates the concentration of the cytokine required for the saturation of the cytokine-binding receptor chain. Second, the binding efficiency of the cytokine/binding chain to the second receptor chain dictates the driving force for the completion of the receptor complex. Third, the expression level of the second receptor chain determines the availability of the second chains, at least in free fluid. All of these three parameters influence the efficiency of IL-4/IL-13 signaling and thereby tune the signal of the immune response in allergic inflammation.

## Author Contributions

IJ planned and wrote the MS.

## Conflict of Interest Statement

The author declares that the research was conducted in the absence of any commercial or financial relationships that could be construed as a potential conflict of interest.
